# Effects of two different paradigms of electrical stimulation exercise on cardio-metabolic risk factors after spinal cord injury. A randomized clinical trial

**DOI:** 10.3389/fneur.2023.1254760

**Published:** 2023-09-22

**Authors:** Ashraf S. Gorgey, Refka E. Khalil, William Carter, Boyd Ballance, Ranjodh Gill, Rehan Khan, Lance Goetz, Timothy Lavis, Adam P. Sima, Robert A. Adler

**Affiliations:** ^1^Spinal Cord Injury and Disorders, Richmond VA Medical Center, Richmond, VA, United States; ^2^Department of Physical Medicine & Rehabilitation, Virginia Commonwealth University, Richmond, VA, United States; ^3^Endocrinology Service, Richmond VA Medical Center, Richmond, VA, United States; ^4^Endocrine Division, School of Medicine, Virginia Commonwealth University, Richmond, VA, United States; ^5^Radiology Service, Richmond VA Medical Center, Richmond, VA, United States; ^6^Department of Biostatistics, School of Medicine, Virginia Commonwealth University, Richmond, VA, United States

**Keywords:** neuromuscular electrical stimulation, functional electrical stimulation, resistance training, spinal cord injury, rehabilitation

## Abstract

**Objective:**

To examine the combined effects of neuromuscular electrical stimulation-resistance training (NMES-RT) and functional electrical stimulation-lower extremity cycling (FES-LEC) compared to passive movement training (PMT) and FES-LEC in adults with SCI on (1) oxygen uptake (VO_2_), insulin sensitivity and glucose disposal in adults with SCI; (2) Metabolic and inflammatory biomarkers; (3) skeletal muscle, intramuscular fat (IMF) and visceral adipose tissue (VAT) cross-sectional areas (CSAs).

**Materials and methods:**

Thirty-three participants with chronic SCI (AIS A-C) were randomized to 24 weeks of NMES-RT + FES or PMT + FES. The NMES-RT + FES group underwent 12 weeks of evoked surface NMES-RT using ankle weights followed by an additional 12 weeks of progressive FES-LEC. The control group, PMT + FES performed 12 weeks of passive leg extension movements followed by an additional 12 weeks of FES-LEC. Measurements were performed at baseline (BL; week 0), post-intervention 1 (P1; week 13) and post-intervention 2 (P2; week 25) and included FES-VO_2_ measurements, insulin sensitivity and glucose effectiveness using the intravenous glucose tolerance test; anthropometrics and whole and regional body composition assessment using dual energy x-ray absorptiometry (DXA) and magnetic resonance imaging to measure muscle, IMF and VAT CSAs.

**Results:**

Twenty-seven participants completed both phases of the study. NMES-RT + FES group showed a trend of a greater VO_2_ peak in P1 [*p* = 0.08; but not in P2 (*p* = 0.25)] compared to PMT + FES. There was a time effect of both groups in leg VO_2_ peak. Neither intervention elicited significant changes in insulin, glucose, or inflammatory biomarkers. There were modest changes in leg lean mass following PMT + FES group. Robust hypertrophy of whole thigh muscle CSA, absolute thigh muscle CSA and knee extensor CSA were noted in the NMES-RT + FES group compared to PMT + FES at P1. PMT + FES resulted in muscle hypertrophy at P2. NMES-RT + FES resulted in a decrease in total VAT CSA at P1.

**Conclusion:**

NMES-RT yielded a greater peak leg VO_2_ and decrease in total VAT compared to PMT. The addition of 12 weeks of FES-LEC in both groups modestly impacted leg VO_2_ peak. The addition of FES-LEC to NMES-RT did not yield additional increases in muscle CSA, suggesting a ceiling effect on signaling pathways following NMES-RT.

**Clinical trial registration:**

identifier NCT02660073.

## 1. Background

Cardio-metabolic risk factors are considered among all-cause mortality in persons with spinal cord injury (SCI). These factors have been well described and characterized by diminished cardiovascular performance, insulin resistance, dyslipidemia and increased visceral adipose tissue (VAT) that leads to central obesity (1, 2). Recent guidelines supported by systematic reviews and randomized clinical trials reported the efficacy of two different approaches of electrical stimulation to train paralyzed muscles in persons with SCI (3–5). The first approach is recognized as functional electrical stimulation (FES) and commonly used for lower extremity cycling (FES-LEC) (4, 6). The second approach, surface neuromuscular electrical stimulation-resistance training (NMES-RT) (7–9), relies on activation of a single muscle group by progressively lifting ankle weights to evoke muscle hypertrophy. Both techniques yield a spectrum of improvements in cardio-metabolic profile in persons with SCI (8–15). Furthermore, a recent systematic review highlighted the superior effect of NMES-RT in inducing skeletal muscle hypertrophy after SCI (16).

In the last three decades, several problems have been identified during applications of FES-LEC. FES-LEC induced premature fatigue of trained muscles resulting in a reduction of torque output and overall cycling performance (17, 18). Premature fatigue affects cycling performance which may interfere with training intensity and subsequently limit cardio-metabolic benefits (19). This may also be explained by a short duty cycle (i.e., on/off time) for each muscle group which results in less tension than required to induce conditioning of the paralyzed muscles (20). As a result, the VO_2_ peak of untrained individuals with SCI may not exceed 0.4 L/min suggesting a very low exercise intensity from using FES-LEC (17). Another concern is that FES-LEC predominately relies on carbohydrate as a source of energy with less reliance on fat (21). Reliance mainly on glycolysis during exercise for 30–60 min is inefficient and may contribute to pre-mature fatigue during FES-LEC (22). After SCI, muscle fiber types transform from slow-oxidative to fast fatigable glycolytic fibers (23); which is accompanied by mitochondrial dysfunction (24). Because of the COVID-19 pandemic, several rehabilitation programs have utilized secure telehealth systems to reduce travel time, waiting lists and risk of hospital acquired infections. However, most persons with SCI do not have access to FES-LEC for home use. Additionally, previous trials reported that adherence dropped remarkably after 8 weeks of home use of expensive FES-LEC ergometers (25). Therefore, it is empirical to provide a rehabilitation approach that can address these limitations of FES-LEC and may serve as an alternative approach in persons with SCI.

Surface NMES-RT has been safely used in home-settings in individuals with chronic SCI (7, 26). In addition, NMES-RT combined with testosterone treatment (TT) resulted in increased fiber cross-sectional area (CSA), citrate synthase a biomarker of mitochondrial density and succinate dehydrogenase in persons with SCI (27). Another study demonstrated increase knee extensor specific tension after 16 weeks of NMES-RT and TT. The peak torque of the trained extensor increased by 48% accompanied with 17% slowness in the rise time (28). Knee extensor muscle group may provide 80% of the driving power during FES-LEC (29). The knee extensor muscle group atrophied by 50% compared to the pre-injury size after SCI (30); which may impact the performance during FES-LEC. A previous randomized clinical trial demonstrated that FES-LEC combined with progressive RT for 12 weeks resulted in greater muscle size and peak torque compared to FES-LEC only in persons with incomplete SCI (31). Therefore, these findings suggest that addition of NMES-RT may attenuate several of the limitations of FES-LEC and potentially enhance the effects of FES-LEC on cardiometabolic risk factors.

The overall objectives of the current trial are to determine the impact of evoking skeletal muscle hypertrophy using surface NMES-RT prior to conducting FES-LEC on oxygen uptake, insulin sensitivity and glucose effectiveness (primary outcome variables) compared to those who underwent passive movement and FES-LEC training only. We hypothesized that 12 weeks of NMES-RT prior to FES-LEC may result in greater skeletal muscle hypertrophy, decreasing IMF and VAT, and further enhance gains in aerobic fitness and insulin sensitivity observed during a subsequent 12-week training of FES-LEC.

## 2. Methods

### 2.1. Study design

A 5 years, 2015–2020, two-site, randomized controlled study was conducted to investigate the efficacy of NMES-RT + FES versus PMT + FES (control group) on cardio-metabolic risk factors after SCI. A detailed study protocol was previously published that highlighted the primary objectives of the work (32). After signing an approved informed consent, each participant underwent a detailed physical examination, including neurological assessment, and International Standards for Neurological Classification of Spinal Cord Injury (ISNCSCI). Using block randomization, participants were randomized into either 12 weeks of NMES-RT followed by 12 weeks of FES-LEC or PMT for 12 weeks followed by 12 weeks of FES-LEC. The entire duration of the study is 27 weeks (3 weeks of measurements and 24 weeks of training). Measurements were conducted at bassline (BL; prior starting any intervention), post-intervention 1 (P1; 12 weeks after intervention) and post-intervention 2 (P2; 24 weeks after intervention). Preliminary results from the current trial were previously published (9, 33).

Thirty-three individuals, with chronic (≥1-year post injury) SCI were randomized into either NMES-RT + FES (*n* = 17) or PMT + FES (*n* = 16; Table 1). Study inclusion and exclusion criteria were previously listed. Briefly, participants were between 18 and 65 years old, men/women, greater than 1-year post SCI, with BMI ≤ 30 kg/m^2^. Participants with motor complete or incomplete C5-L2 level of injury, the American Spinal Injury Association (ASIA) Impairment Scale (AIS) classification A, B, or C were considered for the trial. Participants with pre-existing chronic medical conditions [cardiovascular disease, uncontrolled type II DM, uncontrolled hypertension, insulin dependence, pressures injuries stage 3 or greater, hematocrit above 50%, urinary tract infection, or participants with neck of femur or total body osteoporosis (T-score equal or worse than −2.5 SD) and bone mineral density of distal femur and proximal tibia (less than 0.6 gm/cm^2^) to reduce the likelihood of fracture during training] were excluded from the trial (32).

**Table 1:** Demographic, physical and SCI characteristics of 33 participants who were randomized into 24 weeks of NMES-RT + FES or PMT + FES.

Particip. ID	Group	Sex	Age (yrs.)	Ethne.	Weight (kg)	Height (cm)	BMI (kg/m^2^)	NLI	AIS	TSI (yrs.)	CLASSIF.	Range of lifted weights (lbs)	Total missed visits
001–10,123	PMT + FES	M	48	AA	95.2	183.2	28.4	T4	A	17	Paraplegia	N/A	1
002–10,187	PMT + FES	M	61	C	75.2	182.5	22.6	L1	B	34	Paraplegia	N/A	0
006–10,142	PMT + FES	M	25	AA	60.6	174.5	19.9	C8	A	2	Paraplegia	N/A	8
010–10,177	PMT + FES	M	51	AA	84.6	169.1	29.6	T5	B	9	Paraplegia	N/A	2
011–10,064	PMT + FES	M	30	C	61.8	176.2	19.9	T10	A	5	Paraplegia	N/A	4
016–10,140	PMT + FES	M	21	C	52.6	178.3	16.5	T4	A	7	Paraplegia	N/A	3
017–10,178	PMT + FES	M	36	AA	66.8	179.7	20.7	T5	A	7	Paraplegia	N/A	1
018–10,154	PMT + FES	M	25	AA	52.3	184.2	15.4	C6	C	1	Tetraplegia	N/A	1
022–10,130	PMT + FES	F	51	AA	69.4	164.1	25.8	C6	A	13	Tetraplegia	N/A	2
023–10,113	PMT + FES	M	51	C	83.2	181.3	25.3	T5	A	1.75	Paraplegia	N/A	0
027–10,148	PMT + FES	M	57	C	71.9	182.4	21.6	C5	A	35	Tetraplegia	N/A	0
030–10,019	PMT + FES	M	25	C	58.0	185.4	16.9	C7	B	2	Tetraplegia	N/A	4
033–10,052	PMT + FES	F	46	C	73.8	163.0	27.8	T1	A	8	Paraplegia	N/A	1
036–10,063	PMT + FES	M	57	AA	60.3	167.0	21.6	T11	C	1.5	Paraplegia	N/A	5
038–10,166	PMT + FES	F	32	AA	51.3	151.1	22.5	T4	B	1	Paraplegia	N/A	4
039–10,106	PMT + FES	F	55	C	76.7	166.7	27.6	C5	A	14	Tetraplegia	N/A	5
Mean	16	12 M:4F	41.9	8AA:8C	68.4	174.3	22.6	C5-L1	10 A: 4B:2C	9.9	6 T: 10 P		2.6
SD			13.8		12.8	9.8	4.4	6 T: 10 P		10.8			2.3
003–10,122	NMES-RT + FES	M	34	AA	68.1	182.2	20.5	T12	B	1.5	Paraplegia	0–20	4
004–10,006	NMES-RT + FES	F	50	C	73.4	153.7	31.1	T3	B	29	Paraplegia	0–20	0
005–10,128	NMES-RT + FES	M	53	C	89.7	178.7	28.1	C6	A	26	Tetraplegia	0–0	6
007–10,179	NMES-RT + FES	M	41	C	59.3	172.4	20.0	T4	B	25	Paraplegia	0–22	1
009–10,135	NMES-RT + FES	M	48	C	63.8	174.0	21.1	T8	A	20	Paraplegia	W	6
012–10,181	NMES-RT + FES	M	20	C	83.8	185.9	24.2	C5	B	3	Tetraplegia	0–18	0
014–10,149	NMES-RT + FES	M	27	C	61.0	185.5	17.7	T6	A	4	Paraplegia	0–22	3
015–10,089	NMES-RT + FES	M	41	AA	106.3	173.5	35.3	T11	A	3	Paraplegia	0–2 (R)/ 0–8 (L)	2
019–10,034	NMES-RT + FES	M	33	AA	90.3	172.2	30.5	T8	C	11	Paraplegia	0–22	1
020–10,176	NMES-RT + FES	M	23	C	58.0	178.8	18.1	T6	A	1.58	Paraplegia	0–2	0
024–10,186	NMES-RT + FES	M	44	C	53.5	183.0	16.0	C7	A	13	Tetraplegia	0–0	6
028–10,092	NMES-RT + FES	M	20	AA	45.2	178.7	14.2	C6	B	1.83	Tetraplegia	0–8	4
029–10,180	NMES-RT + FES	M	55	AA	76.4	171.7	25.8	C5	A	30	Tetraplegia	0–12	3
032–10,160	NMES-RT + FES	M	31	AA	70.4	169.4	24.5	T6	A	8	Paraplegia	0-2(L)	6
035–10,098	NMES-RT + FES	F	24	C	72.2	171.3	24.6	T12	C	4	Paraplegia	0–0	2
037–10,039	NMES-RT + FES	M	54	AA	95.0	178.9	29.7	T11	C	4	Paraplegia	0–18	3
040–10,077	NMES-RT + FES	M	46	C	44.8	160.2	17.5	C6	A	20	Tetraplegia	W	3
Mean	17	15 M:2F	37.9	7AA:10C	71.2	174.7	23.5	C5-T12	9A: 5B:3C	12.1	6 T: 11P		2.9
SD			12.3		17.4	8.5	6.0	6 T:11P		10.6			2.2

AA, Afro-American; AIS, American Spinal Cord Injury Impairment Scale; BMI, body mass index; C, Caucasians; CLASSIF, classifications; Ethne, ethnicity; L, Left Leg; N/A, not applicable; NLI, neurological level of injury; P, paraplegia; R, right leg; T, tetraplegia; TSI, time since injury; W, withdrawn.

## 3. Interventions

### 3.1. NMES-resistance training

A video publication providing full details on the NMES-resistance training (NMES-RT) protocol was previously published (34). Briefly, NMES-RT was applied for 12 weeks to the knee extensor muscles via surface electrodes to induce concentric-eccentric actions. Two 8 ×10 cm^2^ adhesive carbon electrodes were placed on the skin over the knee extensor muscle group. After placement of the electrodes, NMES parameters were adjusted at a frequency of 30 Hz, biphasic pulses of 450 μs with interpulse interval of 50 μs and amplitude of current sufficient to evoke knee extension. Training was performed twice weekly, separated by at least 48 h, for 12 weeks with the first week of the NMES-RT performed without ankle weights to ensure that the knee extensor muscles can extend the weight of the lower leg against gravity. The training session consisted of 4 sets of 10 repetitions that were alternated between the right and left knee extensors and separated by 2 min of rest following each set. Once full knee extension was achieved in a sitting position, an increment of 2 lbs. was gradually added per leg on a weekly basis. The increase in ankle weights was only considered when full knee extension was achieved (7, 8, 15).

### 3.2. Passive movement training for the control group

Passive ROM was applied for 12 weeks prior to FES-LEC (9, 32). A member of the research team supported the leg proximal to the ankle joints and moved it from 90 ° knee flexion close to full knee extension. The leg was maintained up for 5 s and returned down for 5 s. The passive movements were repeated in the same fashion described in NMES-RT protocol: 10 reps for the right leg followed by 10 reps for the left leg for total of 4 sets × 10 reps.

### 3.3. Functional electrical stimulation-lower extremity cycling

A video publication providing full details on the functional electrical stimulation-lower extremity cycling (FES-LEC) protocol was previously published (34). FES-LEC was conducted for 12 weeks, twice weekly, for each participant. Rectangular adhesive conductive electrodes were placed on the skin of the knee extensor, hamstrings, and gluteus maximus muscle groups. Pulse frequency was set at 33.3 Hz, pulse duration at 350 μs and resistance was adjusted every 10 min to maintain a speed of 40–45 revolutions per minute (RPM). Resistance of the bike was increased in 0.5 Nm increments per 10-min stage over the course of 12 weeks. The progression in resistance was customized based on the subject’s performance riding the FES-LEC ergometer over 12 weeks. The progression of FES-LEC was previously described in details [see Table 3 in (32)]. The fatigue threshold was set at 18 RPM; if RPM falls below 18 RPM; the bike was set to automatically shift from active to passive cycling (cool-down). During the three-minute cool-down period, participants passively cycled with no electrical stimulation. The cool down period was then followed by 5 min of recovery, during which the participant was still connected to the bike but in a complete resting position while constantly monitoring blood pressure and heart rate.

### 3.4. Dietary recalls

Each participant met with a dietitian at the start of the study and was asked to maintain a weekly 3 to 5-day food dietary log to monitor their caloric and liquid intake for the duration of the study (15, 32). Dietary logs were administered to ensure controlling for the caloric intake and macronutrients. No nutritional advice was given on portion size of the food. However, based on participants’ basal metabolic rate, the dietitian recommended the percentage of macronutrients at 45% carbohydrates, 30% fat and 25% total protein. Dietary logs were analyzed on a weekly basis using a nutritional software package (Nutrition Data System for Research version 2014) under the supervision of a registered dietitian. After the analysis was completed, the average caloric intake (kcal) and percentage macronutrients (carbohydrates, fats and proteins) were calculated, and monthly feedback was provided via phone call (15, 32).

## 4. Measurements

### 4.1. Metabolic profile variables

#### 4.1.1. Leg oxygen uptake using FES-LEC

One week prior to the intervention (week 1), post-intervention 1 (P1; week 14) and post-intervention 2 (P2; week 27), peak oxygen uptake (VO_2_) was measured using a COSMED K4b2 (COSMED USA, Chicago, IL) portable metabolic unit (9, 17). After calibration, subjects were asked to place the mask on their face to monitor oxygen (VO_2_) and carbon dioxide production. A three-minute resting phase allowed the subject to get used to breathing with the mask while on the RT-300 bike. After the resting phase, VO_2_ was measured during a three-minute warm-up phase, the resistance of the bike was gradually increased by 2 Nm every 2 min until fatigue. During testing, the servo motor was tuned off, and the cool-down phase was followed by the recovery phase.

VO_2_ and VCO_2_ were monitored throughout exercise to determine total energy expenditure using the Weir equation. Five minutes of recovery was recorded to determine the efficacy of each intervention on energy expenditure and substrate utilization. Heart rate (via polar HR monitor) was recorded every 30 s and blood pressure (COSMED 740) was recorded before, every 2 min during cycling, and for another 5 min after cycling to ensure full recovery to baseline.

#### 4.1.2. Intravenous glucose tolerance test (primary outcome variables)

A standard intravenous glucose tolerance test (IVGTT) was used to determine insulin sensitivity and glucose effectiveness. Each subject underwent an IVGTT before (BL), and 12 weeks after interventions (P1and P2). After a 10–12-h fast, an indwelling catheter with an intravenous saline drip (0.9% NaCl) was placed. Following 20 min of glucose injection, a bolus of insulin (0.02 U/kg) was injected to determine insulin sensitivity. Plasma glucose was measured by the Autoanalyzer glucose oxidase method and plasma insulin concentrations was determined by commercial radioimmunoassay. The S_I_ (glucose disposal rate per unit of secreted insulin per unit time; i.e., insulin sensitivity) and S_G_ (glucose mediated glucose disposal rate) were calculated from a least-squares fitting of the temporal pattern of glucose and insulin throughout the IVGTT using the MINMOD program (14, 35).

#### 4.1.3. Serum total, free testosterone, IGF, FFA

Total Testosterone measurements were performed by radioimmunoassay after sample extraction and column chromatography. The interassay coefficient of variation (CV) is 12.5% or less for all quality control samples analyzed. Plasma IGF-I and IGFBP-3 concentrations were measured by immunoluminometric assay (Quest Diagnostics, Madison, NJ) and RIA (Diagnostics Systems Laboratories Inc., Webster, TX), respectively. Ten ml of blood was collected from the indwelling venous catheter and lipid profile (HDL-C, LDL-C, total cholesterol, and TG) were determined using standard analyses procedures (15).

#### 4.1.4. Inflammatory biomarkers

Before starting the intravenous glucose tolerance test (IVGTT) and following a 12-h fast, blood was collected from the indwelling venous catheter and CRP, IL-6, TNF-α, and free-fatty acids (FFA) were determined by the Virginia Commonwealth University Clinical Research Center Laboratory using available enzyme-linked immunosorbent assay kits (15).

### 4.2. Body composition

#### 4.2.1. Body mass index and anthropometrics

Each participant was asked to empty their bladder and then propel onto a wheelchair weighing scale to evaluate weight in kg. The wheelchair was measured separately, and the difference taken for the final weight. The height of each participant was determined with the subject on his/her right side in the supine position. Two smooth wooden boards were placed at the participant’s head and heels and the distance between them determined the height in nearest cm. The Body mass index (BMI) (Kg/m^2^) was calculated as weight (kg)/height^2^ (m^2^). Anthropometrics were determined in duplicate by identifying the narrowest region of the trunk from sitting and lying positions. After normal expiration, a tape measure was used around the participant’s trunk to measure waist circumference (WC) (35–37).

#### 4.2.2. Dual energy x-ray absorptiometry (DXA)

Total body and regional (lumbar spine, proximal femur, and forearm) DXA scans were performed using a GE Lunar iDXA (Lunar Inc., Madison, WI) bone densitometer DXA was used to measure body composition in SCI individuals, specifically regional and total fat mass (FM) and fat-free mass (FFM). at the Hunter Holmes VAMC hospital. All scans were performed and analyzed using Lunar software version 10.5. After scanning, total and regional % FM and FFM were determined using DXA software. The longitudinal precision of total and regional body composition using DXA as well as the percentage error compared to the gold-standard body composition technique were previously determined in persons with SCI (34, 35). Body composition assessment of the upper extremity serves as an internal control for repeated measure longitudinal trial (34).

#### 4.2.3. Magnetic resonance imaging

Skeletal muscle CSAs were determined before (baseline), and twice after 12-week interventions (post-intervention 1 and post-intervention 2) using a 1.5 Tesla GE magnet (9, 15). Transaxial images, 0.8 cm thick and 1.6 cm apart, were taken from the hip joint to the knee joint (thigh) and from knee to the ankle (leg) using the whole-body coil. T1-weighted imaging was performed using a fast spin-echo sequence to capture visceral fat images. To measure visceral adipose tissue (VAT) and subcutaneous adipose tissue (SAT), transverse slices (0.8 cm thickness) are acquired every 0.4 cm gap from the xyphoid process to the femoral heads. Images were acquired in a series of two stacks with L4-L5 used as a separating point. Participants were asked to take a deep breath in and hold their breath for 10–15 s to reduce the respiratory-motion artifact associated with magnetic resonance imaging (MRI) for the abdominal region (36, 37). For analysis purpose, VAT-SAT slices were classified according to the distribution across different anatomical regions into VAT_L-K_ or SAT_L-K_ [between liver (L) and kidneys (K)], VAT_K-Um_ or SAT_K-Um_ [between the kidneys and umbilicus], VAT_IC-F_ or SAT _IC-F_ [between iliac crests and femoral heads] and VAT_total_ or SAT_total_ [the average of the entire multi-axial slices from the liver to femoral heads]. Finally, VAT: SAT ratio was calculated across different anatomical regions as well as for the total trunk region.

### 4.3. Statistical analyses

Using a block randomization, a 2 × 2 design was developed in which participants were matched based on level of injury (tetraplegia vs. paraplegia) and time since injury (less versus more than 10 years). Randomization was conducted using n-query computer program at the baseline prior enrollment in the trial. A Supplementary Table S1 was included to highlight the entire procedure for randomization for the trial. Allocation into either PMT + FES or NMES-RT + FES groups was based in the order of enrollment in the trial.

All data were tested for normality using the Shapiro–Wilk tests. Outliers were detected using normal Q-Q plots at different time points (BL, P1, P2) for each group. If normality was not assumed (*p* < 0.05), the examined variable was then log-transformed before conducting any statistical analyses. Independent T-tests were conducted to examine physical characteristics (age, weight, height, BMI, time since injury) between both groups (NMES-RT + FES and Passive + FES). To account for baseline variabilities on the dependent variables (body composition and metabolic variables), multivariate analysis of covariance (MANCOVA) was conducted to statistically analyze the primary (VO_2_, Si, Sg) and secondary variables of the study. The baseline measurement served as the covariate, both the post-intervention 1 and 2 measurements served as the dependent variables and the group assignments (NMES-RT + FES vs. Passive + FES) served as a fixed factor. If the assumptions of MANCOVA was violated, mixed model analysis of variance (MANOVA) was then used to determine whether there a time effect (baseline, post-int1 and post-int 2), between group effects or interaction. If there is a time effect, repeated measure ANOVA was then used after applying the split data function. Independent *t*-tests were also conducted if the MANOVA revealed an interaction. When appropriate, a Bonferroni post-hoc adjustment for multiple comparisons was performed to control for type II error. Linear regression analyses were used to test the association between body composition variables and different metabolic variables. The study was powered based on preliminaryVO_2_ peak data following NMES-RT and yielded an effect size of 0.432 and a power of 99.82%. Partial eta squared (η^2^*_p_*) measurements were reported for the primary outcome variables. SPSS missed data function was used to estimate missing values for the primary outcome variables (VO_2_, Si and Sg) only when participants completed BL and P1 assessment visits (only for 4 participants). Although 33 participants were enrolled at baseline, statistical analyses were only conducted for 27 participants (82%). The other 6 participants were withdrawn after being randomized at different phases through the trial. Statistical analyses were performed using IBM-SPSS version 29.0 (SPSS, Chicago, IL). Statistical significance was set at alpha level of 0.05 and all values are presented as mean ± SD.

## 5. Results

Originally, 40 participants were enrolled in the trial. Five participants were considered screen failure and 2 participants withdrew immediately after sinigang a consent form without any intervention. Thirty-three participants were enrolled and randomized in the trial in which 6 of them withdrew at different phases of the trial. Of the 6 participants, 4 participants competed P1 in the PMT + FES group and two participants withdrew from the NMES-RT + FES group in weeks 5 and 11 because of problems with transportation and COVID19 pandemic, respectively. Therefore, data analyses were based on the 27 participants who completed the entire study.

**Figure 1:**
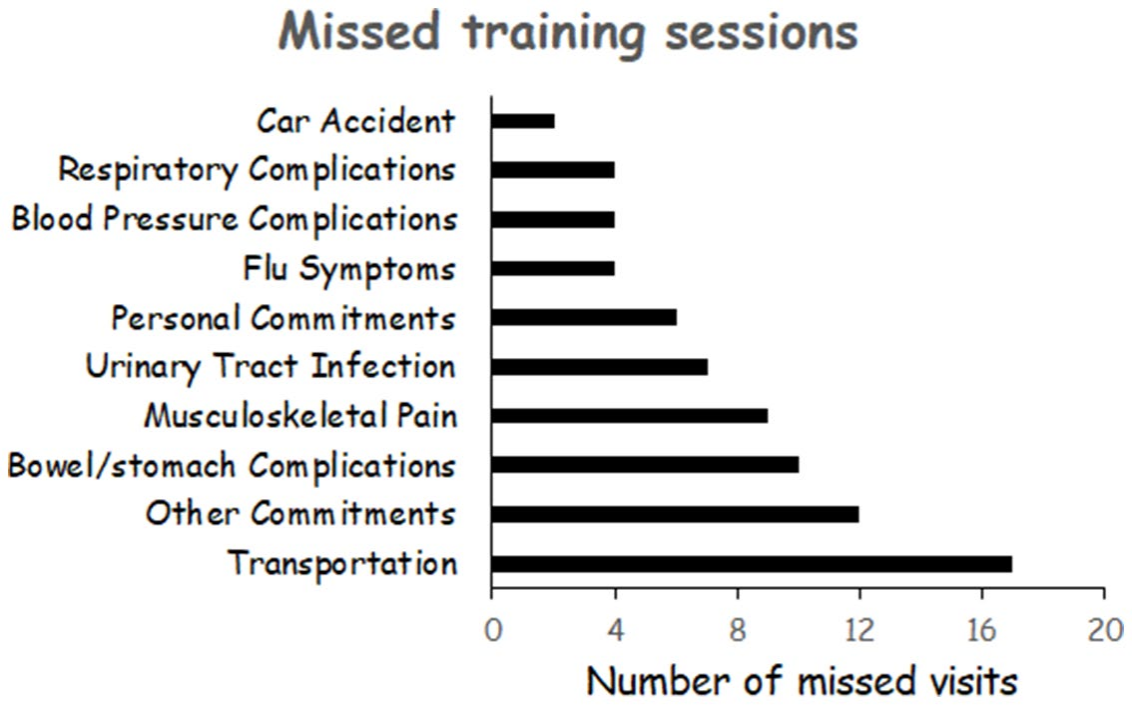
Primary factors that contributed to missed visits across the entire trial for both groups.

Participant demographics and injury characteristics are presented in Table 1. There were no differences in participants’ physical and SCI characteristics between the NMES-RT + FES and PMT + FES groups (*p* > 0.05). Recruitment of the study was discontinued in February 2020 because of the COVID-19 pandemic. Two active participants were asked to discontinue training because of the fear of contracting COVID-19 (ID# 039 and ID# 40). Their data were not included in the trial. Figure 1 illustrates the number of missed visits across the trial and the primary factors that contributed to these missed visits. The total number of missed visits were not different between groups (*p* = 0.63; Table 1). On average, the number of missed visits did not exceed 2 visits before P1 (1.35 ± 1.66) and P2 (1.93 ± 1.77) in the NMES-RT + FES group. On contrary, the average number of missed visits was 0 and 2 before P1 (0.38 ± 0.62) and P2 (2.33 ± 1.91), respectively, in the PMT + FES group.

The average caloric intake and percentage macronutrients are presented in Table 2. Compared to PMT + FES group, NMES-RT + FES group showed a trend of greater % protein intake in P1 (19.5 ± 4.4 vs. 17.2 ± 3.6%, *p* = 0.06). Additionally, there was a trend of lower %fat intake in the NMES-RT + FES compared to PMT + FES group.

**Table 2:** Effects of 24 weeks of PMT + FES compared to NMES-RT + FES on primary and secondary outcome variables of the metabolic profile in persons with spinal cord injury.

		PMT + FES			NMES-RT + FES		
		Baseline	Post-Int1	Post-Int 2	Baseline	Post-Int1	Post-Int 2
Dietary Records & Macronutrients	Caloric intake (kcal)	1,718 ± 857	1,728 ± 759	1,745 ± 692	1,693 ± 623	1,674 ± 403	1,804 ± 547
	%Fat	35 ± 6.0	34 ± 6.0	36.0 ± 5.0	37 ± 7.3	33.0 ± 7.0	33.0 ± 6.0
	%Carbohydrate	46 ± 8.0	48 ± 6.0	46 ± 8.0	42 ± 9.0	47 ± 8.0	47 ± 8.0
	%Protein	18.5 ± 4.0	17 ± 4.0	18 ± 4.0	19 ± 5.0	19.5 ± 4.0	18 ± 4.0
Oxygen uptake (VO_2_)	FES-LEC VO_2_ (l/min)	0.49 ± 0.24 (*n* = 14)	0.48 ± 0.19 (*n* = 14)	0.57 ± 0.27 (*n* = 14)	0.48 ± 0.15 (*n* = 13)	0.58 ± 0.20^#^ (*n* = 13)	0.62 ± 0.23 (*n* = 13)
	Relative VO_2_ (ml/kg/min)	7.7 ± 4.7 (*n* = 14)	7.4 ± 3.6 (*n* = 14)	9.0 ± 5.1 (*n* = 14)	6.5 ± 2.5 (*n* = 13)	8.1 ± 4.0 (*n* = 13)	8.3 ± 4.0 (*n* = 13)
Carbohydrate Profile	Fasting Plasma glucose (mg/dl)	96.6 ± 21.4 (*n* = 14)	91.5 ± 7.0 (*n* = 14)	96.0 ± 22.0 (*n* = 14)	90.0 ± 13.0 (*n* = 12)	90.0 ± 15.0 (*n* = 12)	93.0 ± 16.0 (*n* = 12)
	Fasting Plasma insulin (μU/ml)^L^	6.1 ± 5.0 (*n* = 16)	5.7 ± 3.3 (*n* = 16)	8.2 ± 9.1 (*n* = 16)^M^	7.1 ± 5.7 (*n* = 15)	5.7 ± 3.3 (*n* = 15)	8.3 ± 5.7 (*n* = 15)^M^
	Sg (min-^1^)	0.048 ± 0.08 (*n* = 14)	0.022 ± 0.01 (*n* = 14)	0.024 ± 0.016 (*n* = 14)	0.083 ± 0.23 (*n* = 12)	0.02 ± 0.01 (*n* = 12)	0.021 ± 0.01 (*n* = 12)
	Si^L^	4.4 ± 3.0 (*n* = 11)^O^	6.2 ± 5.0 (*n* = 11)	7.2 ± 6.6 (*n* = 11)^M^	6.6 ± 6.0 (*n* = 13)^O^	5.7 ± 4.9 (*n* = 13)	5.6 ± 5.0 (*n* = 13)^M^
Energy expenditure	BMR (kcal.day^−1^)	1,410 ± 173 (*n* = 13)	1,370 ± 3,170 (*n* = 13)	1,408 ± 118 (*n* = 13)	1,524 ± 211 (*n* = 11)	1,519 ± 138 (*n* = 11)	1,569 ± 234 (*n* = 11)
	BMR.lean mass^−1^ (kcal/g)	0.033 ± 0.004 (*n* = 13)	0.032 ± 0.004 (*n* = 13)	0.032 ± 0.003 (*n* = 13)	0.032 ± 0.004 (*n* = 11)	0.032 ± 0.003 (*n* = 11)	0.033 ± 0.004 (*n* = 11)
	Respiratory exchange ratio (RER)	0.85 ± 0.05 (*n* = 14)	0.85 ± 0.04 (*n* = 15)	0.84 ± 0.07 (*n* = 12)	0.82 ± 0.05 (*n* = 13)	0.84 ± 0.05 (*n* = 13)	0.86 ± 0.03 (*n* = 12)
Lipid profile	TG (mg/dl)^L^	95.5 ± 46.0 (*n* = 14)	103.0 ± 59.0 (*n* = 14)	92.0 ± 47 (*n* = 14)	110 ± 59 (*n* = 12)	97 ± 41 (*n* = 12)	94 ± 44.5 (*n* = 12)
	LDL-C (mg/dl)	86 ± 20 (*n* = 14)	82 ± 23 (*n* = 14)	79 ± 26 (*n* = 14)	112 ± 32 (*n* = 12)	117 ± 40^*^ (*n* = 12)	103 ± 28^*^ (*n* = 12)
	HDL-C (mg/dl)	42 ± 10 (*n* = 14)	42 ± 10 (*n* = 14)	44 ± 11 (*n* = 14)	39 ± 7 (*n* = 12)	38 ± 6 (*n* = 12)	38 ± 7 (n = 12)
	Non-HDL-C (mg/dl)	105 ± 23 (*n* = 14)	103 ± 25 (*n* = 14)	97 ± 30 (*n* = 14)	134 ± 48 (*n* = 12)	137 ± 43^*?^ (*n* = 12)	122 ± 31^*?^ (*n* = 12)
	Total cholesterol (TC; mg/dl)	147 ± 23 (*n* = 14)	145 ± 21 (*n* = 14)	142 ± 29 (*n* = 14)	173 ± 39 (*n* = 12)	175 ± 43 (*n* = 12)	160 ± 30 (*n* = 12)
	TC: HDL-C	3.7 ± 1.3 (*n* = 14)	3.7 ± 1.4 (*n* = 14)	3.4 ± 1.3^*?^ (*n* = 14)	4.5 ± 1.2 (*n* = 12)	4.6 ± 1.2 (*n* = 12)	4.3 ± 1.1 (*n* = 12)
Anabolic profile	Serum testosterone (ng/dl)	291 ± 214 (*n* = 14)	296 ± 222 (*n* = 14)	284 ± 233 (*n* = 14)	364 ± 215 (*n* = 11)	376 ± 197 (*n* = 11)	381 ± 214 (*n* = 11)
	IGF-1^L^ (ng/ml)	138 ± 54 (*n* = 10)	128 ± 52 (*n* = 10)	134 ± 59 (*n* = 10)	148 ± 57 (*n* = 9)	147 ± 47 (*n* = 9)	138 ± 49 (*n* = 9)
	IGBP-3 (ng/dl)	1,897 ± 424 (*n* = 10)	1,850 ± 392 (*n* = 10)	1,822 ± 368 (*n* = 10)	1,840 ± 389 (*n* = 8)	1,854 ± 409 (*n* = 8)	1,779 ± 330 (*n* = 8)
Inflammatory biomarkers	TNFα (pg/ml)	21.5 ± 4.1 (*n* = 10)	24.0 ± 4.5 (*n* = 10)	22.6 ± 2.5 (*n* = 10)	22.5 ± 4.0 (*n* = 9)	22.4 ± 5.1 (*n* = 9)	21.3 ± 5.7 (*n* = 9)
	IL6 (pg/ml)^L^	6.9 ± 12.0 (*n* = 9)	7.0 ± 9.6 (*n* = 9)	4.9 ± 5.0 (*n* = 9)	5.7 ± 4.7 (*n* = 5)	12.0 ± 21 (*n* = 5)	410.0 ± 15.0 (*n* = 5)
	CRP (ng/ml)^L,X^	15,946 ± 27,023 (*n* = 10)	12,523 ± 12,827 (*n* = 10)	5,459 ± 8,607 (*n* = 10)	14,704 ± 13,055 (*n* = 10)	10,868 ± 14,688 (*n* = 10)	19,502 ± 39,703 (*n* = 10)
	FFA (μg/ml)^L^	7.1 ± 6.1 (*n* = 10)	5.4 ± 4.4 (*n* = 10)	5.0 ± 4.7^*^ (*n* = 10)	5.0 ± 1.7 (*n* = 8)	4.7 ± 1.7 (*n* = 8)	3.6 ± 2.1^*^ (*n* = 8)

^#^A trend toward difference in VO_2_ between groups; L: data were logged transformed before running statistical analysis, because the data did not meet the assumption of normality; O: outliers resulted in omission of the data. ^*^Pairwise comparison difference indicating a time effect within a group. ^*?^A trend towards pairwise comparisons. ^x^Interaction between groups, *p* = 0.048. ^M^Missing values were considered in Post-Int 2.

### 5.1. Power and resistance of FES-LEC

Power and resistance data of FES-LEC were not normally distributed and did not meet the assumption of normality after being log-transformed. Non-parametric Mann–Whitney U was used to analyze the difference between group. The PMT + FES group induced a greater resistance at P1 compared to the NMES-RT + FES group (5.1 ± 4.6 vs. 3.6 ± 1.9 Nm, *p* = 0.027). NMES-RT + FES showed a trend of increase in power (6.0 ± 3.5 to 13 ± 12.5 W, *p* = 0.08) and resistance (31 ± 1.1 to 6.6 ± 6.5 Nm, *p* = 0.06) in P2 compared to BL.

#### 5.1.1. Metabolic profile variables

##### 5.1.1.1. Effects of NMES-RT + FES vs. PMT + FES on FES-LEC VO_2_ peak

The data for VO_2_ peak (Table 2) was normally distributed (*p* = 0.086–0.23). MANCOVA demonstrated that NMES-RT + FES (*n* = 13) had a trend towards a greater FES-LEC VO_2_ peak in P1(*p* = 0.08; η^2^*_p_ = 0.12*) but not in P2 (*p* = 0.25) compared to PMT + FES (*n* = 14). Mixed model ANOVA revealed that there was a time effect (*p* = 0.001; η^2^*_p_* = 0.23). Pairwise comparisons revealed that that PMT + FES elicited a trend in VO_2_ peak following P2 compared to P1 (*p* = 0.078); whereas NMES-RT + FES resulted in changes in VO_2_ peak following P2 compared to BL (*p* = 0.0005) but not after P1 (*p* = 0.11).

There was no difference in relative VO_2_ between groups at P1 (*p* = 0.14) and at P2 (*p* = 0.57). Repeated MANOVA showed a time effect (*p* = 0.007; η^2^*_p_* = 0.18) in relative VO_2_. Pairwise comparisons demonstrated that PMT + FES resulted in difference following P2 compared to P1 (*p* = 0.042) but not compared to BL (*p* = 0.16). Pairwise comparisons showed that NMES-RT + FES increased (*p* = 0.034) relative VO_2_ in P2 compared to baseline (Table 2).

##### 5.1.1.2. Effects of NMES-RT + FES vs. PMT + FES on metabolic profile

There was no difference between groups in fasting plasma glucose at either P1 (*p* = 0.93) or P2 (*p* = 0.80) or fasting plasma insulin at P1 (*p* = 0.9) or P2 (*p* = 0.6). Additionally, there was no difference between both groups on Sg [P1; *p* = 0.3 and P2; *p* = 0.42] and log-transformed values of Si at [P1; *p* = 0.23 and P2; *p* = 0.3]. Finally, log-transformed values of HOMA-B and HOMA-IR were not different between groups (Table 2).

There were no differences in BMR between both groups at P1 (*p* = 0.27) and P2 (*p* = 0.87). Basal metabolic rate adjusted to total body lean mass was not different between groups at P1 (*p* = 0.9) and P2 (*p* = 0.5). Similarly, respiratory exchange ratio (RER) did not yield differences between both groups at P1 (*p* = 0.3) and P2 (*p* = 0.8).

##### 5.1.1.3. Effects of NMES-RT + FES vs. PMT + FES on lipid profile

There were no differences between groups in TG, LDL-C, HDL-C, TC after P1 (*p* = 0.37; *p* = 0.36; *p* = 0.7; *p* = 0.53; *p* = 0.4, respectively) or P2 (*p* = 0.6; *p* = 0.62, *p* = 0.18, *p* = 0.7, *p* = 0.7, respectively; Table 2). There was time effect in LDL-C (*p* = 0.02; η^2^*_p_ = 0.15*) within groups. Pairwise comparison indicated that NMES-RT + FES resulted in LDL-C reduction in P2 compared to P1 (12%, *p* = 0.031) but not compared to BL (*p* = 0.38). Non-HDL-C showed a time effect (*p* = 0.023; η^2^*_p_* = 0.15); pairwise comparison indicated that there was a trend (*p* = 0.07) in P2 compared to P1 following NMES-RT + FES but not following PMT + FES (Table 2). There was a trend of a within group effect in TC (*p* = 0.07; η^2^*_p_* = 0.10). Finally, there was a time effect in TC: HDL-C ratio (*p = 0.01; η^2^*_p_ = 0.17) as well as a trend of between group effect (*p* = 0.08). Pairwise comparisons indicated a trend (*p* = 0.07) in P2 compared to BL following PMT + FES (Table 2).

##### 5.1.1.4. Effects of NMES-RT + FES vs. PMT + FES on anabolic biomarkers

Anabolic biomarkers were not analyzed for the last 8 participants (4 participants per group) because of funding constraints. There were no differences in the anabolic biomarkers (serum testosterone, IGF-1 and IGFBP-3) between the two intervention groups (Table 2).

##### 5.1.1.5. Effects of NMES-RT + FES vs. PMT + FES on inflammatory biomarkers

Inflammatory biomarkers were not analyzed for the last 8 participants (4 participants per group) because of funding constraints. Data were logged transformed for IL6, CRP and FFA before running statistical analyses. There were no differences in the inflammatory biomarkers between the two groups. There was interaction between both groups on log-transformed values of CRP (*p* = 0.048; η^2^*_p_* = 0.16). There was time effect in log-transformed FFA (*p* = 0.031; η^2^*_p_* = 0.20) within groups (Table 2).

#### 5.1.2. Body composition variables

##### 5.1.2.1. Anthropometrics

The interventions had no effects on anthropometric variables as demonstrated in Table 3.

**Table 3:** Effects of 24 weeks of PMT + FES compared to NMES-RT + FES on body composition variables in persons with spinal cord injury.

		PMT + FES-LEC			NMES-RT + FES		
		Baseline	Post-Int1	Post-Int 2	Baseline	Post-Int1	Post-Int 2
Anthropometrics	Supine WC	81 ± 13.4 (*n* = 14)	81.5 ± 13.0 (*n* = 14)	83.0 ± 15 (*n* = 14)	86 ± 14.0 (*n* = 12)	85.5 ± 15.0 (*n* = 12)	86.4 ± 13 (*n* = 12)
	Supine AC	81 ± 14.0 (*n* = 14)	81 ± 15.0 (*n* = 14)	82.0 ± 16 (*n* = 14)	87.5 ± 15 (*n* = 13)	88 ± 17.0 (*n* = 13)	87.5 ± 16.5 (*n* = 13)
	Supine Hip circumference	94 ± 12.0 (*n* = 14)	95 ± 13.0 (*n* = 14)	96.0 ± 12 (*n* = 14)	98 ± 10 (*n* = 13)	99 ± 10 (*n* = 13)	100 ± 0.0 (*n* = 13)
	Waist to Hip ratio	0.86 ± 0.06 (*n* = 14)	0.85 ± 0.08 (*n* = 14)	0.86 ± 0.06 (*n* = 14)	0.88 ± 0.08 (*n* = 13)	0.87 ± 0.08 (*n* = 13)	0.87 ± 0.06 (*n* = 13)
	Supine Thigh circumference	46 ± 6.0 (*n* = 14)	47 ± 7.0 (*n* = 14)	48.0 ± 7.0 (*n* = 14)	50 ± 10 (*n* = 13)	50.5 ± 8.0 (*n* = 13)	50 ± 08.0 (*n* = 13)
	Seated Calf circumference	30 ± 3.0 (*n* = 14)	30.5 ± 3.0 (*n* = 14)	30.0 ± 3.1 (*n* = 14)	32.0 ± 4.0 (*n* = 13)	32 ± 4.1 (*n* = 13)	32 ± 4.0 (*n* = 13)
Body composition- DXA							
Upper Extremity	Fat mass (g)	2,270 ± 1,422 (*n* = 14)	2,225 ± 1,221 (*n* = 14)	2,146 ± 1,277 (*n* = 14)	2,231 ± 1,182 (*n* = 13)	2,247 ± 1,062 (*n* = 13)	2,247 ± 1,190 (*n* = 13)
	%Fat mass	25.5 ± 15 (*n* = 14)	26.3 ± 14 (*n* = 14)	25.5 ± 15 (*n* = 14)	24.5 ± 9 (*n* = 13)	25.6 ± 11.0 (*n* = 13)	26.5 ± 11.0 (*n* = 13)
	Lean mass (g)	6,167 ± 2,358 (*n* = 14)	5,956 ± 2,318 (*n* = 14)	6,023 ± 2,356 (*n* = 14)	6,739 ± 1,480 (*n* = 13)	6,222 ± 1,776 (*n* = 13)	6,306 ± 1,716 (*n* = 13)
	BMC (g)	437 ± 114 (*n* = 14)	436 ± 120 (*n* = 14)	437 ± 120 (*n* = 14)	445 ± 78 (*n* = 13)	432 ± 81 (*n* = 13)	432 ± 82 (*n* = 13)
	Total mass (kg)	8.8 ± 2.7 (*n* = 14)	8.6 ± 2.7 (*n* = 14)	8.6 ± 2.7 (*n* = 14)	9.5 ± 2.1 (*n* = 13)	8.9 ± 2.2 (*n* = 13)	9.2 ± 2.3 (*n* = 13)
Lower extremity	Fat mass (g)	6,562 ± 3,355 (*n* = 14)	6,877 ± 3,575 (*n* = 14)	6,674 ± 3,871 (*n* = 14)	7,711 ± 3,870 (*n* = 13)	7,420 ± 3,625 (*n* = 13)	7,905 ± 3,574 (*n* = 13)
	%Fat mass	25.5 ± 15 (*n* = 14)	33 ± 13 (*n* = 14)	31 ± 13 (*n* = 14)	32.0 ± 9.0 (*n* = 13)	32.0 ± 9.4 (*n* = 13)	32.5 ± 10.0 (*n* = 13)
	Lean mass (g)	12,643± 2,622 (*n* = 14)	12,342± 2,687 (*n* = 14)	13,104 ± 2,519 (*n* = 14)	14,351 ± 2,561 (*n* = 13)	14,183± 3,207 (*n* = 13)	14,854± 3,140 (*n* = 13)
	BMC (g)	789.5 ± 217	788 ± 207	759 ± 203	823 ± 194	791 ± 215^#^	811 ± 223
	Total mass (kg)	20 ± 3.6	20 ± 3.8	20.5 ± 4.2	23 ± 5.5	22.4 ± 5.8	23.6 ± 5.4
Trunk	Fat mass (g)	11,246 ± 7,154 (*n* = 14)	11,627 ± 7,563 (*n* = 14)	11,475 ± 7,632 (*n* = 14)	13,773 ± 7,707 (*n* = 13)	14,382 ± 8,253 (*n* = 13)	13,971 ± 7,736 (*n* = 13)
	%Fat mass	31.4 ± 16.0 (*n* = 14)	32.0 ± 16.0 (*n* = 14)	31.3 ± 17.0 (*n* = 14)	34.3 ± 12.0 (*n* = 13)	35.1 ± 14.0 (*n* = 13)	35.4 ± 14.0 (*n* = 13)
	Lean mass (g)	20,835 ± 3,643 (*n* = 14)	20,783 ± 3,413 (*n* = 14)	21,089 ± 3,116 (*n* = 14)	22,757 ± 2,831 (*n* = 13)	22,676 ± 1,776 (*n* = 13)	21,974 ± 2,846 (*n* = 13)
	BMC (g)	824 ± 219 (*n* = 14)	808 ± 234 (*n* = 14)	832 ± 231 (*n* = 14)	904 ± 221 (*n* = 13)	879 ± 221 (*n* = 13)	864 ± 167 (*n* = 13)
	Total mass (kg)	33 ± 8 (*n* = 14)	33 ± 8 (*n* = 14)	33 ± 8 (*n* = 14)	37 ± 10 (*n* = 13)	38 ± 10 (*n* = 13)	37 ± 9 (*n* = 13)
Total	Fat mass (g)	21,133 ± 11,334 (*n* = 14)	21,927 ± 12,006 (*n* = 14)	21,465 ± 12,577 (*n* = 14)	24,890 ± 11,551 (*n* = 13)	25,222 ± 12,058 (*n* = 13)	25,357 ± 11,953 (*n* = 13)
	%Fat mass	30 ± 13 (*n* = 14)	30.7 ± 13 (*n* = 14)	29.6 ± 14 (*n* = 14)	31.5 ± 9.4 (*n* = 13)	32 ± 11 (*n* = 13)	32.3 ± 11.0 (*n* = 13)
	Lean mass (g)	43,629 ± 8,167 (*n* = 14)	43,282 ± 7,114 (*n* = 14)	44,181 ± 7,226 (*n* = 14)	47,644 ± 5,959 (*n* = 13)	47,464 ± 6,089 (*n* = 13)	46,892 ± 6,808 (*n* = 13)
	BMC (g)	2,724 ± 467 (*n* = 14)	2,713 ± 458 (*n* = 14)	2,679 ± 470 (*n* = 14)	2,781 ± 466 (*n* = 13)	2,710 ± 481 (*n* = 13)	2,690 ± 466 (*n* = 13)
	Total mass (kg)	67.5 ± 12.7 (*n* = 14)	68 ± 13 (*n* = 14)	68 ± 14 (*n* = 14)	75 ± 16 (*n* = 13)	75 ± 16 (*n* = 13)	75 ± 15 (*n* = 13)

^#^Difference between groups.

##### 5.1.2.2. DXA

Repeated measure analysis indicated that there is a significant decline in upper extremity lean mass (*p* = 0.009). Pairwise comparison showed that there was 433 g decline (6.4%) in the NMES-RT + FES (*p* = 0.012). There was a total decline in total mass of the upper extremity mass (*p* = 0.04); pairwise comparison indicated that there was a trend of decline in total mass (*p* = 0.08) following P1 in the NMES-RT + FES (Table 3).

Mixed model analysis indicated increases (*p* = 0.012) in leg lean mass (g) in the PMT + FES group. Pairwise comparison showed a 0.76 kg increase in P 2 compared to P1 (6%, *p* = 0.041). MANCOVA revealed significant difference in leg bone mineral content (*p* = 0.038) between the groups following P1 (Table 3).

##### 5.1.2.3. Magnetic resonance imaging

Table 4 highlights the changes in muscle and IMF CSAs following PMT + FES and NMES-RT + FES. Muscle CSA was presented in the forms of whole or absolute CSA (i.e., after subtracting IMF) for whole thigh and knee extensor muscle group. Table 4 denoted the changes in muscle hypertrophy in the whole muscle CSA and knee extensors, respectively.

**Table 4:** Effects of 24 weeks of PMT + FES compared to NMES-RT + FES on muscle CSA and intramuscular fat (IMF) in persons with spinal cord injury.

		PMT + FES						NMES-RT + FES					
		Right			Left			Right			Left		
		BL	P1	P2	BL	P1	P2	BL	P1	P2	BL	P1	P2
Whole Muscle CSA	Proximal	101 ± 22 (*n* = 15)	97 ± 22 (*n* = 12)	107 ± 23^*^ (*n* = 13)	98 ± 22 (*n* = 14)	90 ± 25 (*n* = 12)	113 ± 25^**^ (*n* = 11)	95 ± 33 (*n* = 15)	114 ± 32^##**^ (*n* = 14)	117 ± 34^*^ (*n* = 12)	96 ± 33 (*n* = 15)	113 ± 30^#**^ (*n* = 14)	122 ± 31^**^ (*n* = 12)
	Middle	89 ± 22 (*n* = 15)	87 ± 25 (*n* = 12)	96 ± 24^*^ (*n* = 13)	88 ± 23 (*n* = 14)	89 ± 27 (*n* = 12)	102 ± 27^**^ (*n* = 11)	85 ± 33 (*n* = 15)	104 ± 31^##**^ (*n* = 14)	109 ± 33^**^ (*n* = 12)	87 ± 32 (*n* = 15)	104 ± 31^**^ (*n* = 14)	113 ± 31^**^ (*n* = 12)
	Distal	70 ± 17 (*n* = 15)	71 ± 21 (*n* = 12)	81 ± 21^**^ (*n* = 13)	72 ± 19 (*n* = 13)	72 ± 22 (*n* = 12)	83 ± 22^**^ (*n* = 11)	70 ± 26 (*n* = 15)	81 ± 22^#*^ (*n* = 14)	86 ± 23^*^ (*n* = 12)	70 ± 25 (*n* = 15)	81 ± 22^*^ (*n* = 14)	83 ± 21^*^ (*n* = 11)
	Average	87 ± 19 (*n* = 15)	86 ± 22 (*n* = 12)	94 ± 22^*^ (*n* = 13)	88 ± 21 (*n* = 14)	87 ± 24 (*n* = 12)	101 ± 24^*^ (*n* = 11)	84 ± 30 (*n* = 15)	100 ± 27^##**^ (*n* = 14)	104 ± 29^**^ (*n* = 12)	85 ± 29 (*n* = 15)	100 ± 26^#**^ (*n* = 14)	108 ± 28^**^ (*n* = 12)
ABS Whole Muscle CSA	Proximal	84 ± 23 (*n* = 15)	76 ± 26 (*n* = 12)	84 ± 27 (*n* = 13)	84 ± 26 (*n* = 14)	79 ± 25 (*n* = 12)	90 ± 24 (*n* = 12)	84 ± 29 (*n* = 15)	101 ± 31^#*^ (*n* = 14)	102 ± 30^*^ (*n* = 12)	84 ± 28 (*n* = 15)	99 ± 26^#*^ (*n* = 14)	106 ± 27^*^ (*n* = 12)
	Middle	73 ± 22 (*n* = 15)	68 ± 32 (*n* = 12)	74 ± 23 (*n* = 13)	73 ± 23 (*n* = 14)	69 ± 22 (*n* = 12)	79 ± 24 (*n* = 12)	72 ± 27 (*n* = 15)	86 ± 30^*^ (*n* = 14)	92 ± 29^*^ (*n* = 12)	72 ± 27 (*n* = 15)	86 ± 25^#*^ (*n* = 14)	90 ± 25 (*n* = 12)
	Distal	53 ± 17 (*n* = 15)	48 ± 17 (*n* = 12)	51 ± 22 (*n* = 13)	60 ± 20 (*n* = 14)	47 ± 20^*^ (*n* = 12)	55 ± 23 (*n* = 12)	52 ± 19 (*n* = 15)	56 ± 19 (*n* = 14)	63 ± 20 (*n* = 12)	49 ± 18 (*n* = 15)	57 ± 19^#^ (*n* = 14)	60 ± 18 (*n* = 11)
	Average	71 ± 19 (*n* = 15)	65 ± 21 (*n* = 12)	71 ± 23 (*n* = 13)	73 ± 20 (*n* = 14)	67 ± 20 (*n* = 12)	76 ± 22 (*n* = 12)	70 ± 24 (*n* = 15)	82 ± 24^#^ (*n* = 14)	86 ± 24^*^ (*n* = 12)	69 ± 23 (*n* = 15)	82 ± 21^#*^ (*n* = 14)	85 ± 22^*^ (*n* = 11)
KE Muscle CSA	Proximal	45 ± 13 (*n* = 15)	41 ± 14 (*n* = 12)	50 ± 15^*^ (*n* = 13)	43 ± 14 (*n* = 14)	42 ± 15 (*n* = 12)	52 ± 16^**^ (*n* = 12)	40 ± 13 (*n* = 15)	53 ± 14^##**^ (*n* = 14)	53 ± 15^**^ (*n* = 12)	40 ± 13 (*n* = 15)	52 ± 14^# **^ (*n* = 14)	55 ± 14^**^ (*n* = 12)
	Middle	41 ± 13 (*n* = 15)	40 ± 16 (*n* = 12)	46 ± 15^*^ (*n* = 13)	40 ± 14 (*n* = 14)	40 ± 16 (*n* = 12)	49 ± 17^**^ (*n* = 12)	38 ± 14 (*n* = 15)	50 ± 14^##**^ (*n* = 14)	52 ± 15^**^ (*n* = 12)	38 ± 13 (*n* = 15)	49 ± 15^**^ (*n* = 14)	53 ± 15^**^ (*n* = 12)
	Distal	32 ± 9 (*n* = 15)	33 ± 12 (*n* = 12)	36 ± 12^*^ (*n* = 13)	32 ± 10 (*n* = 14)	33 ± 13 (*n* = 12)	39 ± 13^**^ (*n* = 12)	30 ± 11 (*n* = 15)	38 ± 10^#**^ (*n* = 14)	40 ± 10^*^ (*n* = 12)	30 ± 12 (*n* = 15)	37 ± 10.5^#*^ (*n* = 14)	36 ± 9^#*^ (*n* = 11)
	Average	40 ± 11 (*n* = 15)	39 ± 13 (*n* = 12)	45 ± 14^*^ (*n* = 13)	39 ± 12.3 (*n* = 14)	39 ± 14.4 (*n* = 12)	47 ± 15^**^ (*n* = 12)	37 ± 12 (*n* = 15)	47 ± 11^##**^ (*n* = 14)	49 ± 13^**^ (*n* = 12)	36 ± 12 (*n* = 15)	46 ± 12^**^ (*n* = 14)	49 ± 13.3^**^ (*n* = 11)
ABS KE Muscle CSA	Proximal	40 ± 13 (*n* = 15)	34 ± 15 (*n* = 12)	39 ± 18 (*n* = 13)	38 ± 15 (*n* = 14)	36 ± 14 (*n* = 12)	43 ± 17 (*n* = 12)	38 ± 13 (*n* = 15)	47 ± 15^#*^ (*n* = 14)	47 ± 15 (*n* = 12)	36 ± 12 (*n* = 15)	47 ± 14^#**^ (*n* = 14)	49 ± 15^*^ (*n* = 12)
	Middle	36 ± 12 (*n* = 15)	32 ± 14 (*n* = 12)	36 ± 15 (*n* = 13)	35 ± 14 (*n* = 14)	34 ± 14 (*n* = 12)	39 ± 16 (*n* = 12)	34 ± 12 (*n* = 15)	41 ± 16 (*n* = 14)	45 ± 14 (*n* = 12)	32 ± 12 (*n* = 15)	41 ± 14^#*^ (*n* = 14)	43 ± 14^*^ (*n* = 11)
	Distal	26 ± 8 (*n* = 15)	23 ± 10 (*n* = 12)	25 ± 13 (*n* = 13)	25 ± 11 (*n* = 14)	22 ± 12 (*n* = 12)	27 ± 14 (*n* = 12)	24 ± 9 (*n* = 15)	27 ± 12 (*n* = 14)	29 ± 11 (*n* = 12)	22 ± 9 (*n* = 15)	27 ± 12 (*n* = 14)	27 ± 10 (*n* = 11)
	Average	34 ± 10 (*n* = 15)	30 ± 13 (*n* = 12)	34 ± 15 (*n* = 13)	34 ± 13 (*n* = 14)	31 ± 13 (*n* = 12)	37 ± 15 (*n* = 12)	32 ± 11 (*n* = 15)	39 ± 13 (*n* = 14)	41 ± 12 (*n* = 12)	30 ± 10 (*n* = 15)	39 ± 12^#*^ (*n* = 14)	39 ± 12^*^ (*n* = 11)
Whole Thigh IMF	Proximal	16 ± 12 (*n* = 15)	21 ± 18 (*n* = 12)	24 ± 22 (*n* = 13)	15 ± 11 (*n* = 14)	18.6 ± 13.6 (*n* = 12)	21 ± 17 (*n* = 12)	10 ± 8 (*n* = 15)	13 ± 9 (*n* = 14)	14 ± 10 (*n* = 12)	12 ± 12 (*n* = 15)	12 ± 9 (*n* = 14)	16 ± 14 (*n* = 12)
	Middle	16 ± 11 (*n* = 15)	19 ± 13 (*n* = 12)	22 ± 20^*^ (*n* = 13)	14 ± 8 (*n* = 14)	18 ± 12 (*n* = 12)	21 ± 17 (*n* = 12)	13 ± 10 (*n* = 15)	18 ± 14 (*n* = 14)	17 ± 13 (*n* = 12)	15 ± 11 (*n* = 15)	16 ± 10 (*n* = 14)	17 ± 14 (*n* = 12)
	Distal	18 ± 12 (*n* = 15)	24 ± 14 (*n* = 12)	25 ± 21 (*n* = 13)	19 ± 11 (*n* = 14)	26 ± 15 (*n* = 12)	26 ± 19 (*n* = 12)	18 ± 13 (*n* = 15)	25 ± 16 (*n* = 14)	22 ± 17 (*n* = 12)	20 ± 14 (*n* = 15)	23 ± 10 (*n* = 14)	23 ± 13 (*n* = 11)
	Average	16 ± 11 (*n* = 15)	21 ± 14 (*n* = 12)	23 ± 20^*^ (*n* = 13)	16 ± 8 (*n* = 14)	20 ± 13 (*n* = 12)	22 ± 17 (*n* = 12)	14 ± 10 (*n* = 15)	19 ± 13 (*n* = 14)	18 ± 12 (*n* = 12)	15.7 ± 11 (*n* = 15)	17 ± 9^#^ (*n* = 15)	18 ± 13 (*n* = 12)
Whole Thigh % IMF	Proximal	17 ± 13 (*n* = 15)	22 ± 18 (*n* = 12)	19 ± 16 (*n* = 12)	17 ± 14 (*n* = 14)	19 ± 15 (*n* = 12)	19 ± 16 (*n* = 12)	11 ± 7 (*n* = 15)	12 ± 9 (*n* = 14)	13 ± 9 (*n* = 12)	13 ± 8 (*n* = 15)	11 ± 8 (*n* = 14)	13 ± 10 (*n* = 12)
	Middle	18 ± 13 (*n* = 15)	22 ± 15 (*n* = 12)	20 ± 15 (*n* = 12)	17 ± 11 (*n* = 14)	21 ± 14 (*n* = 12)	20 ± 15 (*n* = 12)	15 ± 8 (*n* = 15)	18 ± 13 (*n* = 14)	15 ± 11 (*n* = 12)	17 ± 11 (*n* = 15)	16 ± 8 (*n* = 14)	15 ± 11 (*n* = 12)
	Distal	25 ± 16 (*n* = 15)	33 ± 16 (*n* = 12)	29 ± 19 (*n* = 12)	24 ± 14 (*n* = 14)	36 ± 21 (*n* = 12)	32 ± 21 (*n* = 12)	25 ± 12 (*n* = 15)	30 ± 17 (*n* = 14)	25 ± 17 (*n* = 12)	28 ± 14 (*n* = 15)	30 ± 11(*n* = 14)	27 ± 14 (*n* = 11)
	Average	19 ± 13 (*n* = 15)	25 ± 15 (*n* = 12)	22 ± 15 (*n* = 12)	19 ± 10 (*n* = 14)	24 ± 14 (*n* = 12)	23 ± 16 (*n* = 12)	17 ± 8 (*n* = 15)	19 ± 12 (*n* = 14)	17 ± 10 (*n* = 12)	19 ± 10 (*n* = 15)	18 ± 8 (*n* = 14)	17 ± 11 (*n* = 12)
KE IMF	Proximal	5.5 ± 4.8 (*n* = 15)	8.4 ± 11 (*n* = 12)	10.9 ± 13.2^*^ (*n* = 13)	4.8 ± 4.0 (*n* = 14)	6.2 ± 6.1 (*n* = 12)	8.9 ± 10.4 (*n* = 12)	2.9 ± 2.1 (*n* = 15)	5.6 ± 5.4 (*n* = 14)	6.2 ± 5.7 (*n* = 12)	4.5 ± 5.1 (*n* = 15)	4.5 ± 3.5^#^ (*n* = 14)	6.1 ± 6.8 (*n* = 12)
	Middle	5.0 ± 4.8 (*n* = 15)	7.7 ± 8.7 (*n* = 12)	10.7 ± 12.9^*^ (*n* = 13)	5.2 ± 3.7 (*n* = 14)	6.2 ± 5.6 (*n* = 12)	9.1 ± 9.4 (*n* = 12)	4.5 ± 3.9 (*n* = 15)	9.2 ± 9.5 (*n* = 14)	7.6 ± 7.4 (*n* = 12)	6.0 ± 5.8 (*n* = 15)	7.4 ± 5.4 (*n* = 14)	7.4 ± 7 (*n* = 12)
	Distal	5.4 ± 4.5 (*n* = 15)	9.9 ± 8.7 (*n* = 12)	11.3 ± 12.9 (*n* = 12)	6.2 ± 4.2 (*n* = 14)	10.3 ± 8.2 (*n* = 12)	11.7 ± 10.5 (*n* = 12)	5.9 ± 4.6 (*n* = 15)	11.1 ± 9.6 (*n* = 14)	9.8 ± 9.4 (*n* = 12)	8.3 ± 7.2 (*n* = 15)	10.0 ± 5.6^#^ (*n* = 14)	9.1 ± 6.1 (*n* = 11)
	Average	5.2 ± 4.5 (*n* = 15)	8.5 ± 9.1 (*n* = 12)	10.9 ± 12.5^*^ (*n* = 13)	5.4 ± 3.4 (*n* = 14)	7.4 ± 5.8 (*n* = 12)	9.8 ± 9.9 (*n* = 12)	4.4 ± 3.2 (*n* = 15)	8.6 ± 8 (*n* = 14)	7.9 ± 6.8 (*n* = 12)	6.2 ± 5.7 (*n* = 15)	7.3 ± 4.8 (*n* = 14)	7.2 ± 6 (*n* = 12)
KE % IMF	Proximal	12.4 ± 10.3 (*n* = 15)	19.6 ± 21.1 (*n* = 12)	22.2 ± 23.5^*^ (*n* = 13)	13 ± 12.3 (*n* = 14)	15.2 ± 14 (*n* = 12)	17.7 ± 19.9 (*n* = 12)	7.8 ± 6.6 (*n* = 15)	11.8 ± 12.8 (*n* = 14)	12.3 ± 13.2 (*n* = 12)	11.0 ± 11.6 (*n* = 15)	9.2 ± 7.6 (*n* = 14)	11.8 ± 13.6 (*n* = 12)
	Middle	11.8 ± 9.9 (*n* = 15)	18.7 ± 15.4 (*n* = 12)	21.9 ± 20.8 (*n* = 13)	14.0 ± 10.5 (*n* = 14)	15.8 ± 11.6 (*n* = 12)	18.8 ± 17.3 (*n* = 12)	11.9 ± 9.0 (*n* = 15)	18.9 ± 20.2 (*n* = 14)	14.5 ± 14.1 (*n* = 12)	15.9 ± 14.1 (*n* = 15	15.9 ± 10.8 (*n* = 14)	15.2 ± 14.2 (*n* = 12)
	Distal	17.2 ± 12.5 (*n* = 15)	28.7 ± 17.8 (*n* = 12)	31.3 ± 24.1 (*n* = 13)	20.9 ± 15.2 (*n* = 14)	32.2 ± 22.8 (*n* = 12)	30.7 ± 23.6 (*n* = 12)	19.5 ± 11.2 (*n* = 15)	29.4 ± 24.6 (*n* = 14)	23.7 ± 22.2 (*n* = 12)	25.0 ± 17.3 (*n* = 15)	29.5 ± 16.5 (*n* = 14)	26.8 ± 20.1^#^ (*n* = 11)
	Average	13.3 ± 10.0 (*n* = 15)	21.7 ± 17.0 (*n* = 12)	24.3 ± 21.8* (*n* = 13)	15.3 ± 9.9 (*n* = 14)	19.9 ± 13.1 (*n* = 12)	21.6 ± 19.8 (*n* = 12)	12.8 ± 7.6 (*n* = 15)	19.7 ± 18.7 (*n* = 14)	16.7 ± 13.8 (*n* = 12)	17.1 ± 13.3 (*n* = 15)	17.7 ± 10.9 (*n* = 14)	17.0 ± 14.4 (*n* = 12)

Proximal CSA represent the first 4 MRI slices starting with the first one just at the inferior border of the gluteus maximus muscle; middle CSA represents the 4 slices of the mid-thigh; distal CSA represents the average of the 4–6 slices towards the knee joints. Average CSA represents the average of the entire 12–14 slices per right or left leg. ^##^Between group differences *p* < 0.001. ^#^Between group differences *p* < 0.001–*p* ≤ 0.049. ^**^Time effect within group, *p* < 0.001. ^*^Time effect within group, 0.001 < *p* ≤ 0.043.

The entire data for VAT, SAT and VAT: SAT ratio did not meet the assumption of normality and had to be log-transformed (Table 5). There was a trend of 16% decrease in VAT_L-K_ following NMES-RT compared to baseline (*p* = 0.054). VAT_K-Um_ and VAT_IC-F_ showed a trend of lower CSA in the NMES-RT + FES compared to PMT-FES following P1 (*p* = 0.06) and P2 (*p* = 0.084), respectively. Finally, VAT_total_ was 26.7 and14.2% lower in the NMES-RT + FES compared to PMT-FES following P1 (*p* = 0.023) and P2 (*p* = 0.050), respectively.

**Table 5:** Effects of 24 weeks of PMT + FES compared to NMES-RT + FES on central obesity variables (VAT, SAT, VAT: SAT ratio) in persons with spinal cord injury.

		PMT + FES-LEC			NMES-RT + FES		
		Baseline	Post-Int1	Post-Int 2	Baseline	Post-Int1	Post-Int 2
Visceral adipose tissue (VAT)^L^	VAT_L-K_ (cm^2^)	68.2 ± 74 (*n* = 14)	67 ± 78 (*n* = 12)^!^	73.3 ± 86 (*n* = 13)	55.2 ± 59 (*n* = 13)	46.2 ± 57 (*n* = 12)	58.1 ± 60 (*n* = 11)
	VAT_k-Um_ (cm^2^)	89.2 ± 81 (*n* = 14)	94 ± 98 (*n* = 12)	93.4 ± 92 (*n* = 13)	80.2 ± 90 (*n* = 13)	69 ± 84^#?^ (*n* = 12)	87.5 ± 97 (*n* = 11)
	VAT_IC-F_ (cm^2^)	54.2 ± 40 (*n* = 14)	53 ± 38 (*n* = 12)	58.6 ± 53 (*n* = 12)	52.8 ± 40 (*n* = 13)	41.4 ± 27 (*n* = 12)	46.6 ± 34^#?^ (*n* = 11)
	VATtotal (cm^2^)	70 ± 60 (*n* = 14)	71.8 ± 68 (*n* = 12)	75.4 ± 73 (*n* = 13)	62.1 ± 61 (*n* = 13)	52.6 ± 57^#^ (*n* = 12)	64.7 ± 66^#^ (*n* = 11)
Subcutaneous adipose tissue (SAT^)L^	SAT_L-K_ (cm^2^)	76.7 ± 61 (*n* = 14)	69.8 ± 57 (*n* = 12)	81.7 ± 72 (*n* = 13)	111.8 ± 92 (*n* = 14)	96.6 ± 81 (*n* = 13)	104.1 ± 78 (*n* = 12)
	SAT_k-Um_ (cm^2^)	143.3 ± 102 (*n* = 14)	136.3 ± 105 (*n* = 12)	147.5 ± 115 (*n* = 13)	193.7 ± 139 (*n* = 14)	181.9 ± 138 (*n* = 13)	191.8 ± 132 (*n* = 12)
	SAT_IC-F_ (cm^2^)	166.5 ± 116 (*n* = 14)	161.7 ± 129 (*n* = 12)	146.3 ± 113* (*n* = 12)	196 ± 124 (*n* = 14)	191.4 ± 138 (*n* = 13)	191.1 ± 136 (*n* = 12)
	SATtotal (cm^2^)	130.1 ± 94 (*n* = 14)	125.2 ± 99 (*n* = 12)	128.8 ± 98 (*n* = 13)	167.7 ± 119 (*n* = 14)	160.3 ± 121 (*n* = 13)	164 ± 113 (*n* = 12)
VAT:SAT ratio^L^	VAT:SAT_L-K_	0.75 ± 0.3 (*n* = 13)	0.77 ± 0.3 (*n* = 11)	0.8 ± 0.5 (*n* = 12)	0.87 ± 0.7 (*n* = 14)	0.91 ± 0.8 (*n* = 13)	0.71 ± 0.5 (*n* = 11)
	VAT: SAT_k-Um_	0.6 ± 0.3 (*n* = 13)	0.6 ± 0.4 (*n* = 11)	0.65 ± 0.4 (*n* = 12)	0.6 ± 0.5 (*n* = 14)	0.6 ± 0.5 (*n* = 13)	0.53 ± 0.4 (*n* = 11)
	VAT: SAT_IC-F_	0.33 ± 0.2 (*n* = 13)	0.4 ± 0.2 (*n* = 11)	0.4 ± 0.2 (*n* = 11)	0.33 ± 0.2 (*n* = 14)	0.33 ± 0.2 (*n* = 13)	0.33 ± 0.2 (*n* = 11)^#?^
	VAT:SATtotal	0.55 ± 0.3 (*n* = 13)	0.57 ± 0.3 (*n* = 11)	0.6 ± 0.3 (*n* = 12)	0.6 ± 0.4 (*n* = 14)	0.61 ± 0.5 (*n* = 13)	0.57 ± 0.4 (*n* = 11)

^L^Entire data of VAT, SAT, and VAT: SAT ratio were logged transformed for failing to meet the assumption of normality. ^#^Difference between groups (*p* = 0.023–0.05); #?, a trend of between group difference (*p* = 0.06–0.084). ^*^Difference within PMT + FES group (*p* = 0.018). Pairwise comparison suggested difference between P2 and P1 timepoints (*p* = 0.077). L, liver; K, kidneys; IC, iliac crests; F, femoral heads. ^!^MRI scan of one of the participants was not analyzed because interference of the intrathecal baclofen with the field of view of VAT and SAT.

SAT_IC-F_ decreased in the PMT + FES (*p* = 0.018) but not in the NMES-RT + FES group. Pairwise comparisons showed a trend between P2 and P1 (*p* = 0.077) in the PMT-FES group. Finally, there was no changes in the VAT: SAT ratio between both groups.

## 6. Discussion

Several important findings were noted in the current study that are likely to expand our knowledge about the interaction or complementary effects between NMES-RT and FES-LEC. The addition of 12 weeks of FES-LEC following 12 weeks of NMES-RT did not result in additional increase in muscle size. There was an increase in muscle mass after adding 12 weeks of FES-LEC to PMT; however, it was obviously non-significantly greater following NMES-RT. The addition of FES-LEC resulted in recognized gains in power and resistance only in P2 in the NMES-RT + FES; however, the gains in both variables was only noted following P1in the PMT + FES group. Similar to our recent findings (9), NMES-RT managed to increase leg VO_2_ peak compared to PMT; however, the addition of FES-LEC resulted in increasing VO_2_ and relative VO_2_ in P2 compared to P1 in both groups. It is interesting to note that NMES-RT + FES resulted in a 12% decrease in the LDL-C level as well as total trunk VAT CSA. Finally, based on the current findings, home-based training may overcome several of the barriers that emerged during the course of the training, such as missing visits and study discontinuation as a result of the COVID-19 pandemic.

### 6.1. Significance and rationale of the work

Recent studies and guidelines have recommended both aerobic and resistance training to evoke muscle hypertrophy, strength or increasing aerobic capacity, respectively, in persons with SCI (3, 4, 31). We aimed to evoke muscle hypertrophy prior application of FES-LEC training to attenuate several of its existing limitations and to maximize the benefits on cardio-metabolic variables (18, 29). The addition of progressive FES-LEC training as described in this protocol following NMES-RT did not evoke further muscle hypertrophy (see below). However, we demonstrated increased muscle strength as measured by the resistance and power of the FES ergometer bike. The current findings suggest a clear dissociation in musculoskeletal adaptations versus neuromuscular adaptations in the current trial. Previous research indicated that neuromuscular adaptations via increasing neural drive commonly precede muscle hypertrophy (38–40); especially when resistance training is applied for a short period of 4–6 weeks (41). Based on the current findings, progressive FES-LEC enhanced neuromuscular adaptations without evoking muscle hypertrophy in the NMES-RT + FES group. Surprisingly, the PMT + FES group experienced both muscle hypertrophy and increased strength after 12 weeks of just PMT compared to NMES-RT; suggesting a training specificity. It is possible to speculate the evoking muscle hypertrophy may have attenuated the recognized effects of FES-LEC on muscle strength in the NMES-RT + FES compared to the PMT + FES group following P1. Previous work indicated that when aerobic training (AT) preceded RT, the performance of RT was diminished up to 8 h in the muscles that were involved in aerobic training (42). A previous meta-analysis concluded that concurrent AT and RT may attenuate gains in explosive strength; however, the report stressed the need for AT and RT to improve physical fitness and health (43).

### 6.2. Muscle hypertrophy is attenuated in the NMES-RT + FES group

Based on the current report, there is a hiking effect on the signaling pathway involved in evoking muscle hypertrophy in the NMES-RT group. Following 12 weeks of NMES-RT, the hypertrophy signaling pathway attained a ceiling effect. We and others have noted the abundance of protein following either NMES-RT or FES-LEC (26, 44, 45). The paralyzed muscles have intact signaling pathway that can be upregulated when the appropriate stimulation pattern is delivered (44, 45). We have recently studied the primary predictors of muscle hypertrophy between high and low responders with SCI (33). We noted that high responders may experience great Akt protein expression with concomitant increase IGFBP-3 without a recognized changes in circulating IGF-1. Furthermore, mRNA analysis revealed upregulation in IRS-1, Akt, mTOR with concomitant downregulation in myostatin, MurF-1 and PDK4 compared to the low responders (33). Therefore, changing the stimulation paradigm from NMES-RT to FES-LEC did not trigger upregulation or downregulation or signaling pathways to evoke additional muscle hypertrophy. On contrary, the addition of FES-LES to PMT resulted in muscle hypertrophy; however, it deemed less comparable to NMES-RT.

### 6.3. Effects of training on cardio-metabolic risk factors

The current findings support recognized benefits of training on cardio-metabolic risk factors (1). The noticeable change was recognized in VO_2_ peak and predominantly in the NMES-RT + FES group. Furthermore, the NMES-RT + FES resulted in improvement in the LDL-C profile and decrease in total VAT CSA. We have previously demonstrated a 14% increase in FES-LEC VO_2_ peak (i.e., leg VO_2_ peak) following 12–16 weeks of NMES-RT compared to PMT (9). The increases in whole thigh muscle and knee extensor muscle CSAs were associated with increase in VO_2_ peak (9). A recent randomized clinical trial showed that in 76 adolescents adding RT to either moderate continuous AT or to high intensity AT resulted in increasing VO_2_ peak by 4.4 and 5.5%, respectively (46). In the current trial, we noticed 12% increase in VO_2_ peak in adults with SCI. The difference in VO_2_ between groups was non-significantly noted in P1 but was further enhanced in P2 especially in the NMES-RT + FES group. Although statistically different, it is still unclear the clinical implications of these findings for the SCI population. Another randomized clinical trial recommended combination exercise of RT and AT for 5 days per week compared to either RT or AT only in improvement of cardio-respiratory fitness in overweight and obese individuals (47). Additional benefits included decrease in VAT CSA and LDL-C profile. The association of VAT to cardio-metabolic risk factors have been well studied and this has been shown to mediated via increasing inflammatory cytokines and negatively impacting circulating testosterone and mitochondrial activity (48).

The question that remains to be addressed is whether the preceding NMES-RT attenuated the effects of 12 weeks FES-LEC on cardio-metabolic outcomes. Several trials demonstrated the efficacy of FES-LEC on enhancing the cardio-metabolic profile (10, 11, 14). Training drives improvement in cardio-metabolic health is primarily mediated by increasing muscle mass (9) and accompanied with increased mitochondrial density and activity after SCI (27). This will result in subsequent increase in fatty acid oxidation and hence increase insulin sensitivity and enhanced metabolic flexibility. The decrease in VAT CSA as well as LDL-C following NMES-RT + FES supported previous findings. However, the addition of FES-LEC to NMES-RT did not induce additional muscle hypertrophy. This can be explained by possible antagonistic physiological adaptations of AT and RT; which may interfere with each other when the two types of training are performed serially (49). The combination of both RT and aerobic training has been shown to be superior in weight loss to either intervention alone in obese elderly able-bodied persons (49).

### 6.4. Limitations

Several of the current findings were trended towards statistical insignificance. Spinal cord injury is a heterogenous population with wide range of level of injuries and time since injuries. The results of the current trial may serve as important clinical findings towards mitigating cardio-metabolic risk factors after SCI. Contrary, statistical differences in lean mass and BMC may not be of clinical significance and are considered within the error of repeated measures as previously highlighted (34). It is possible that the frequency of training (2x per week) of FES-LEC was ineffective in enhancing cardio-metabolic benefits. Previous trials recommended a frequency of 3x per week. Gater et al. recently demonstrated that 5x per week for 16 weeks of FES-LEC resulted in decreasing percentage body fat in 6 individuals with motor complete SCI (35). We chose a frequency of 2x per week to increase adherence and compliance. Dolbow et al. previously indicated that the adherence following 8 weeks home use of FES-LEC decreased from 72 to 63% (25). We originally powered the study based on VO_2_ change to recruit 48 individuals with SCI (24 per group); however, the 5-year trial resulted only in 33 participants. The small sample size may have possibly impacted the overall findings on the primary outcome variables. Additionally, the COVID-19 pandemic resulted in early discontinuation of the study. Similar to other studies and pre-planned design, the IVGTT was performed 5–7 days following the last training session. It is possible that a shorter window of 36–48 h might have better demonstrated training effects. In addition, unreported changes in dietary habits may have influenced or masked the effects of training on Sg and Si.

## 7. Summary/Conclusion

In conclusion, this is the first randomized clinical trial that examined the effects of evoking muscle hypertrophy via NMES-RT on maximizing the benefits of FES-LEC on cardio-metabolic risk factors in persons with chronic SCI. The use of FES-LEC following 12 weeks of NMES-RT modestly influence cardio-metabolic risk factors and evoked additional muscle hypertrophy as hypothesized. The findings support that VO_2_ peak is the primary factor that appears to be responsive to both training paradigms especially following NMES-RT + FES. The evidence supports the notion that both NMES-RT and FES-LEC may have different training effects on musculoskeletal and neuromuscular adaptions. Evoking muscle hypertrophy may attenuate the elicited neuromuscular adaptations during FES-LEC. Neuromuscular adaptations are further enhanced by FES-LEC suggesting a training specificity. Additionally, there is further mitigation of cardio-metabolic risk factors as noted by improvement in the lipid profile and decrease in VAT after NMES-RT. The inclusion of PMT did not impact any of the examined cardio-metabolic outcomes. We believe that compared to the expensive FES-LEC ergometers, NMES-RT may provide an alternative, simple and cheap rehabilitation approach either in clinical settings or for home-use that may overcome transportation problems, a primary impediment to utilization of proven interventions.

## Data availability statement

The raw data supporting the conclusions of this article will be made available by the authors, without undue reservation after obtaining necessary approvals from Richmond Inst. for Veterans Research.

## Ethics statement

The studies involving humans were approved by Richmond Inst. for Veterans Research IRB. The studies were conducted in accordance with the local legislation and institutional requirements. The participants provided their written informed consent to participate in this study.

## Author contributions

AG: Conceptualization, Data curation, Formal analysis, Funding acquisition, Investigation, Methodology, Project administration, Resources, Supervision, Writing – original draft, Writing – review & editing. RK: Data curation, Supervision, Writing – review & editing. WC: Conceptualization, Data curation, Methodology, Writing – review & editing. BB: Writing – review & editing. RG: Conceptualization, Data curation, Methodology, Supervision, Writing – review & editing. RK: Data curation, Investigation, Methodology, Writing – review & editing. LG: Conceptualization, Data curation, Investigation, Writing – review & editing. TL: Conceptualization, Investigation, Supervision, Writing – original draft. AS: Formal analysis, Investigation, Methodology, Writing – review & editing. RA: Conceptualization, Data curation, Funding acquisition, Investigation, Supervision, Writing – original draft, Writing – review & editing.

## Funding

The author(s) declare financial support was received for the research, authorship, and/or publication of this article. This study was supported by the DoD-CDMRP (W81XWH-14-SCIRP-CTA). The funding agents have nothing to do with the design of the study and data collection, analysis, and interpretation of data and in writing the manuscript should be declared.

## Conflict of interest

The authors declare that the research was conducted in the absence of any commercial or financial relationships that could be construed as a potential conflict of interest.

## Publisher’s note

All claims expressed in this article are solely those of the authors and do not necessarily represent those of their affiliated organizations, or those of the publisher, the editors and the reviewers. Any product that may be evaluated in this article, or claim that may be made by its manufacturer, is not guaranteed or endorsed by the publisher.

## Abbreviations

ASIA: American Spinal Injury Association (ASIA)
AIS: ASIA Impairment Scale
BL: baseline
BMI: body mass index
CSA: cross-sectional area
CRP: C-reactive protein
DXA: dual energy x-ray absorptiometry
FES: functional electrical stimulation
FES-LES: functional electrical stimulation-lower extremity cycling
FM: fat mass
ISNCSCI: International Standards for Neurological Classification of Spinal Cord Injury
IMF: intramuscular fat
IGF-1: insulin growth factors-1
IGFBP-3: insulin growth factors binding protein-3
IVGTT: Intravenous Glucose Tolerance Test
MRI: magnetic resonance imaging
NMES: neuromuscular electrical stimulation
NMES-RT: neuromuscular electrical stimulation-resistance training
PMT: passive movement training
P1: post-intervention 1
P2: post-intervention 2
RPM: revolution per minute
SAT: subcutaneous adipose tissue
SCI: spinal cord injury
TT: testosterone treatment
VAT: visceral adipose tissue
VO_2_: oxygen uptake
