# Supplementary material for: Effects of two different paradigms of electrical stimulation exercise on cardio-metabolic risk factors after spinal cord injury. A randomized clinical trial
Source: Front Neurol. 2023 Sep 22;14:1254760. doi: 10.3389/fneur.2023.1254760 (PMC10556465; doi:10.3389/fneur.2023.1254760)
Supplement: Supplementary file 1 [file Data_Sheet_1.PDF]

Allocation table highlighting block randomization based on time since injury and severity of injury in PMT+FES and NMES-RT+FES groups

		Tetraplegia less than 10 years			Paraplegia less than 10 years			Tetraplegia more than 10 years			Paraplegia more than 10 years		
	Status	Patient Enroll	ID	Assignment	Patient	ID	Assignment	Patient	ID	Assignment	Patient	ID	Assignment
Enrolled	Completed	1	10154	PMT+ FES	1	10122	NMES-RT + FES	1	10128	NMES-RT + FES	1	10123	PMT+ FES
Enrolled	Completed	2	10181	NMES-RT + FES	2	10142	PMT+ FES	2	10130	PMT+ FES	2	10187	PMT+ FES
Enrolled	Completed	3	10019	PMT + FES	3	10177	PMT+ FES	3	10148	PMT+ FES	3	10006	NMES-RT + FES
Enrolled	Completed	4	10092	NMES-RT + FES	4	10064	PMT+ FES	4	10186	NMES-RT + FES	4	10179	NMES-RT + FES
Enrolled	Completed	5	10161	PMT + FES	5	10149	NMES-RT + FES	5	10180	NMES-RT + FES	5	10135	NMES-RT + FES
Enrolled	Withdrawn after P1	6	10091	NMES-RT + FES	6	10089	NMES-RT + FES	6	10106	PMT+ FES	6	10026	PMT+ FES
Enrolled	Completed	7	10165	NMES-RT + FES	7	10140	PMT+ FES	7	10077	NMES-RT + FES	7	10178	PMT+ FES
Enrolled	Screen Failure	8	10144	PMT+ FES	8	10176	NMES-RT + FES	8	10129	PMT+ FES	8	10034	NMES-RT + FES
Enrolled	Withdrawn 5 weeks	9	10099	PMT + FES	9	10163	NMES-RT + FES	9	10125	NMES-RT + FES	9	10120	PMT+ FES
Enrolled	Completed	10	10189	NMES-RT + FES	10	10113	PMT+ FES	10	10126	PMT+ FES	10	10152	NMES-RT + FES
Enrolled	Completed	11	10182	NMES-RT + FES	11	10097	PMT+ FES	11	10170	NMES-RT + FES	11	10086	NMES-RT + FES
Enrolled	Completed	12	10192	PMT + FES	12	10160	NMES-RT + FES	12	10112	PMT+ FES	12	10070	PMT+ FES
Enrolled	Screen Failure	13	10131	PMT+ FES	13	10052	PMT+ FES	13	10040	NMES-RT + FES	13	10158	PMT+ FES
Enrolled	Completed	14	10172	NMES-RT + FES	14	10098	NMES-RT + FES	14	10020	PMT+ FES	14	10065	NMES-RT + FES
Enrolled	Completed	15	10095	PMT + FES	15	10039	NMES-RT + FES	15	10190	NMES-RT + FES	15	10009	PMT+ FES
Enrolled	Completed	16	10073	NMES-RT + FES	16	10063	PMT+ FES	16	10082	PMT+ FES	16	10107	NMES-RT + FES
Enrolled	Completed	17	10051	PMT+ FES	17	10055	NMES-RT + FES	17	10038	PMT+ FES	17	10036	NMES-RT + FES
Enrolled	Completed	18	10029	NMES-RT + FES	18	10166	PMT+ FES	18	10118	NMES-RT + FES	18	10102	PMT+ FES
Enrolled	Completed	19	10037	PMT+ FES	19	10023	PMT+ FES	19	10132	PMT+ FES	19	10105	NMES-RT + FES
Enrolled	Completed	20	10047	NMES-RT + FES	20	10115	NMES-RT + FES	20	10012	NMES-RT + FES	20	10079	PMT+ FES
Enrolled	Early Withdrawal without intervention	21	10053	NMES-RT + FES	21	10191	NMES-RT + FES	21	10159	NMES-RT + FES	21	10022	NMES-RT + FES
Enrolled	Completed	22	10185	PMT+ FES	22	10133	PMT+ FES	22	10183	PMT+ FES	22	10119	NMES-RT + FES
Enrolled	Withdrawn after P1	23	10050	PMT+ FES	23	10059	NMES-RT + FES	23	10138	NMES-RT + FES	23	10153	PMT+ FES
Enrolled	Completed	24	10017	NMES-RT + FES	24	10143	PMT+ FES	24	10066	PMT+ FES	24	10049	PMT+ FES
Enrolled	Screen Failure	25	10101	PMT+ FES	25	10072	PMT+ FES	25	10093	PMT+ FES	25	10010	NMES-RT + FES
Enrolled	Early Withdrawal without intervention	26	10139	NMES-RT + FES	26	10127	NMES-RT + FES	26	10087	NMES-RT + FES	26	10104	PMT+ FES
Enrolled	Completed	27	10173	NMES-RT + FES	27	10111	NMES-RT + FES	27	10042	PMT+ FES	27	10167	PMT+ FES
Enrolled	Withdrawn after P1	28	10003	NMES-RT + FES	28	10084	PMT+ FES	28	10075	NMES-RT + FES	28	10015	NMES-RT + FES
Enrolled	Completed	29	10188	PMT+ FES	29	10117	NMES-RT + FES	29	10018	PMT+ FES	29	10058	NMES-RT + FES
Enrolled	Completed	30	10083	PMT+ FES	30	10114	PMT+ FES	30	10002	NMES-RT + FES	30	10045	PMT+ FES
Enrolled	Screen Failure	31	10110	PMT+ FES	31	10007	PMT+ FES	31	10027	NMES-RT + FES	31	10109	PMT+ FES
Enrolled	Completed	32	10069	NMES-RT + FES	32	10041	NMES-RT + FES	32	10071	PMT+ FES	32	10021	NMES-RT + FES
Enrolled	Completed	33	10184	NMES-RT + FES	33	10030	NMES-RT + FES	33	10134	PMT+ FES	33	10067	PMT+ FES
Enrolled	Screen Failure	34	10008	PMT+ FES	34	10076	PMT+ FES	34	10136	NMES-RT + FES	34	10103	NMES-RT + FES
Enrolled	Withdrawn after P1	35	10048	NMES-RT + FES	35	10168	NMES-RT + FES	35	10081	NMES-RT + FES	35	10124	PMT+ FES
Enrolled	Completed	36	10175	PMT+ FES	36	10060	PMT+ FES	36	10044	NMES-RT + FES	36	10157	PMT+ FES
Enrolled	Completed	37	10169	PMT+ FES	37	10025	PMT+ FES	37	10068	PMT+ FES	37	10145	NMES-RT + FES
Enrolled	Completed	38	10090	NMES-RT + FES	38	10046	NMES-RT + FES	38	10094	PMT+ FES	38	10147	NMES-RT + FES
Enrolled	Completed	39	10001	NMES-RT + FES	39	10164	PMT+ FES	39	10156	PMT+ FES	39	10100	PMT+ FES
Enrolled	Withdrawn because of COVID	40	10150	PMT+ FES	40	10141	NMES-RT + FES	40	10057	NMES-RT + FES	40	10033	NMES-RT + FES
No Enrollment-COVID		41	10013	PMT+ FES	41	10078	PMT+ FES	41	10146	NMES-RT + FES	41	10056	PMT+ FES
No Enrollment-COVID		42	10085	NMES-RT + FES	42	10121	NMES-RT + FES	42	10096	PMT+ FES	42	10074	NMES-RT + FES
No Enrollment-COVID		43	10028	PMT+ FES	43	10137	NMES-RT + FES	43	10088	NMES-RT + FES	43	10061	NMES-RT + FES
No Enrollment-COVID		44	10043	NMES-RT + FES	44	10116	PMT+ FES	44	10031	PMT+ FES	44	10016	PMT+ FES
No Enrollment-COVID		45	10014	PMT+ FES	45	10054	PMT+ FES	45	10005	NMES-RT + FES	45	10162	NMES-RT + FES
No Enrollment-COVID		46	10080	PMT+ FES	46	10035	NMES-RT + FES	46	10011	PMT+ FES	46	10004	PMT+ FES
No Enrollment-COVID		47	10151	NMES-RT + FES	47	10062	PMT+ FES	47	10032	PMT+ FES	47	10174	NMES-RT + FES
No Enrollment-COVID		48	10171	NMES-RT + FES	48	10108	NMES-RT + FES	48	10024	NMES-RT + FES	48	10155	PMT+ FES
